# A Super-Efficient GSM Triplexer for 5G-Enabled IoT in Sustainable Smart Grid Edge Computing and the Metaverse

**DOI:** 10.3390/s23073775

**Published:** 2023-04-06

**Authors:** Mohammad (Behdad) Jamshidi, Salah I. Yahya, Leila Nouri, Hamed Hashemi-Dezaki, Abbas Rezaei, Muhammad Akmal Chaudhary

**Affiliations:** 1 Faculty of Electrical Engineering, University of West Bohemia, Univerzitní 22, 306 14 Pilsen, Czech Republic; 2 Department of Communication and Computer Engineering, Cihan University-Erbil, Erbil 44001, Iraq; 3 Department of Software Engineering, Faculty of Engineering, Koya University, Koya KOY45, Iraq; 4 Institute of Research and Development, Duy Tan University, Da Nang 550000, Vietnam; 5 School of Engineering & Technology, Duy Tan University, Da Nang 550000, Vietnam; 6 Research and Innovation Center for Electrical Engineering (RICE), Faculty of Electrical Engineering, University of West Bohemia (UWB), 301 00 Pilsen, Czech Republic; 7 Department of Electrical Engineering, Kermanshah University of Technology, Kermanshah 6715685420, Iran; 8 Department of Electrical and Computer Engineering, Ajman University, Ajman P.O. Box 346, United Arab Emirates

**Keywords:** energy, sustainability, complex systems, smart grids, IoT, GSM, triplexer, metaverse, edge computing

## Abstract

Global concerns regarding environmental preservation and energy sustainability have emerged due to the various impacts of constantly increasing energy demands and climate changes. With advancements in smart grid, edge computing, and Metaverse-based technologies, it has become apparent that conventional private power networks are insufficient to meet the demanding requirements of industrial applications. The unique capabilities of 5G, such as numerous connections, high reliability, low latency, and large bandwidth, make it an excellent choice for smart grid services. The 5G network industry will heavily rely on the Internet of Things (IoT) to progress, which will act as a catalyst for the development of the future smart grid. This comprehensive platform will not only include communication infrastructure for smart grid edge computing, but also Metaverse platforms. Therefore, optimizing the IoT is crucial to achieve a sustainable edge computing network. This paper presents the design, fabrication, and evaluation of a super-efficient GSM triplexer for 5G-enabled IoT in sustainable smart grid edge computing and the Metaverse. This component is intended to operate at 0.815/1.58/2.65 GHz for 5G applications. The physical layout of our triplexer is new, and it is presented for the first time in this work. The overall size of our triplexer is only 0.007 λ_g_^2^, which is the smallest compared to the previous works. The proposed triplexer has very low insertion losses of 0.12 dB, 0.09 dB, and 0.42 dB at the first, second, and third channels, respectively. We achieved the minimum insertion losses compared to previous triplexers. Additionally, the common port return losses (RLs) were better than 26 dB at all channels.

## 1. Introduction

Growing global concerns about environmental preservation and energy sustainability have arisen due to the multiple impacts of climate change and the increasing demand for energy. To meet the rigorous requirements of industrial applications, sustainable energy solutions need to be developed. In the last ten years, there has been a significant increase in the number of low-cost, diverse sensors that are connected to the internet in smart cities [1]. These sensors and devices are used in many different industries to gather important data that impact business decisions [2]. Advanced research is being conducted to process the large volume of data that is collected in real-time, and has high variability to extract meaningful insights that meet the needs of various business domains, particularly when it comes to the Metaverse [3,4,5]. With the emergence of the 5G-enabled Internet of Things (IoT) and industrial IoT environments, big data analytics present diverse opportunities to analyze data patterns and generate new results, providing large organizations with the necessary tools to make informed decisions [6,7]. A smart grid, or SG, is a complex system that facilitates the two-way exchange of electricity and data. It uses advanced metering infrastructure and communication technologies to monitor, manage and respond to unexpected changes in the power grid [8]. A paper analyzed the research status of AI and provided future research directions [9]. In addition, a safe decision controller for autonomous driving using deep reinforcement learning in a nondeterministic environment was conducted in another paper [10]. The controller aimed to ensure the safety of the autonomous vehicle by learning optimal actions that minimize the risk of collision with other objects [10].

Microstrip passive devices such as dual-channel diplexers [11], multi-channel diplexers [12], triplexers [13], and multiplexers [14] are widely demanded by modern wireless communication systems. A triplexer is a type of multiplexer which enables a single co-axial cable to run into the feed of three different antennas. It is used in triple-band devices such as power combiners, power dividers, and other wireless transmitter and receiver systems. Meanwhile, the operating frequencies make it suitable for GSM and 5G applications. However, increasing the number of channels and ports makes the design process more complex. Therefore, triplexers are less often reported than diplexers. Some microstrip triplexers have been introduced in [15,16,17,18,19,20,21,22,23,24,25,26,27,28,29]. The common disadvantage of these reported microstrip triplexers is that they occupy large implementation areas, which causes a serious problem. In [15], a switchable microstrip triplexer was designed to operate at 1.2/1.8/2.4 GHz. In [16], a star-junction microstrip topology was used to design a triplexer. In [17], six microstrip asymmetric split-ring resonators were integrated to design a triplexer for wireless applications. The microstrip triplexer in [18] had high isolation and close and narrow channels, but the losses are very high. A common triple-mode resonator has been utilized to obtain a microstrip triplexer with very narrow channels [19]. To design a triplexer, microstrip coupled step impedance open-loop resonators have been used [20]. This triplexer had a large size with very high losses since the physical structure was not well optimized. A microstrip triplexer was proposed in [21] for 4G LTE and IEEE 802.16 WiMAX applications. The designed triplexer in [22] operated at 900 MHz for GSM and 2.4 GHz and 5.5 GHz for WLAN applications. The final layout configuration of this triplexer was somewhat similar to directional couplers. In [23], based on open loop resonators, a microstrip diplexer and triplexer were designed. The interdigital cells were used to obtain a microstrip triplexer [24], which was suitable for GSM and WiMAX applications. The microstrip triplexer in [25] was designed based on quarter-wave resonators. High insertion and return losses (RLs) were the other problems of the presented triplexers in [15,16,17,18,19,20,26,27,29]. Table 1 summarizes the features of the previous triplexers, which includes their basic resonators, applications, advantages, and disadvantages.

None of the mentioned triplexers could reduce the size significantly, while our triplexer has a compact size of 0.007 λ_g_^2^ which is the best compared to the previously reported triplexers. Moreover, having a wide fractional bandwidth (FBW) is an advantage in the design of triplexers. Our triplexer achieves an FBW of 15%, which is the best compared to the previous works. Additionally, the proposed triplexer is a low-loss microstrip device which makes it suitable for energy harvesting applications, while the majority of the previous triplexers had higher insertion losses at all channels than ours.

In this paper, we present a design of a very compact low-loss microstrip triplexer for GSM and 5G applications. The design steps include mathematically analyzing a basic resonator, designing three bandpass filters (BPFs) using the analyzed resonator, and integrating the BPFs. For the utilized resonator, the scattering matrix was calculated. Then, the conditions for reducing the loss and tuning the resonance frequency were obtained simultaneously. Using the presented mathematical analysis, the resonator behavior was determined. Therefore, the proposed structure could be easily optimized. To verify the advantages of this work, a comparison with previously reported microstrip triplexers was set.

In this paper, we make several important contributions in the areas of sustainable smart grid edge computing, metaverse technology, and 5G-enabled IoT.

(1) First, we propose an approach that combines the concepts of sustainable smart grid edge computing and Metaverse technology to achieve a sustainable concept;
(2) Second, we describe how 5G-enabled IoT completes the digitalization process for sustainable smart grids and Metaverses, emphasizing that it is crucial for creating a sustainable infrastructure;
(3) Third, we analyze the requirements for nodes in the edge layer and the edge node and highlight the need for highly efficient IoT operations with 5G. We suggest that achieving this efficiency is essential for the success of the framework;
(4) Finally, we design, manufacture, and measure a new GSM triplexer that is specifically designed for 5G-enabled IoT. A GSM triplexer is a device that separates signals in the radio frequency range, and our device is particularly efficient for 5G-enabled IoT. Despite its compact size, the performance of the proposed triplexer is good as it has very low insertion losses.

Growing global concerns about environmental preservation and energy sustainability have arisen due to the multiple impacts of climate change and the increasing demand for energy. To meet the rigorous requirements of industrial applications, sustainable energy solutions need to be developed. Smart grid technology, which incorporates advanced communication and computing capabilities to optimize energy consumption and distribution, holds promise in achieving this objective. However, traditional private power networks may not suffice to meet the demands of modern industrial applications. Therefore, researchers are investigating innovative technologies such as edge computing and Metaverse-based platforms. The 5G technology is well-suited for smart grid services due to its distinct features such as high reliability, low latency, and large bandwidth. The progress of the 5G network industry heavily relies on the Internet of Things (IoT), which will act as a catalyst for the development of future smart grids. Despite the advantages of these technologies, there are still significant challenges to address, such as the optimization of the IoT for sustainable edge computing networks. To address this challenge, this study presents the design, fabrication, and evaluation of a highly efficient GSM triplexer for 5G-enabled IoT in sustainable smart grid edge computing and the Metaverse. The proposed triplexer is intended to function at 0.815/1.58/2.65 GHz for 5G applications and has the smallest overall size, 0.007 λ_g_^2^, compared to previous works. Additionally, the proposed triplexer exhibits very low insertion losses of 0.12 dB, 0.09 dB, and 0.42 dB at the first, second, and third channels, respectively, and outperforms previous triplexers. Furthermore, the common port return losses (RLs) are better than 26 dB at all channels, indicating the effectiveness of the proposed approach.

This paper is divided into six sections to discuss various aspects of 5G-enabled IoT and GSM triplexer design. Section 2 outlines the framework for 5G-enabled IoT, which is a crucial component in the development of sustainable smart grids and Metaverse platforms. Section 3 presents a detailed analysis of the structure of the proposed GSM triplexer specifically designed for 5G-enabled IoT. In Section 4, the results and comparisons of the GSM triplexer are presented, highlighting its efficiency and performance. Section 5 discusses the findings and their implications, including the potential for further improvements in 5G-enabled IoT and sustainable smart grid edge computing. Finally, the paper concludes by summarizing the key findings and emphasizing the importance of optimizing the IoT for achieving a sustainable edge computing network.

## 2. A Framework for 5G-Enabled IoT

As smart grid technology has advanced, it has become apparent that traditional private power networks are no longer sufficient to meet the demanding requirements of industrial applications [2]. This is particularly true in terms of bandwidth, end-to-end latency, and reliability. To address this problem, experts have suggested that integrating 5G mobile communication technology with various industries could be the way forward [30]. The unique capabilities of 5G, such as its ability to support large bandwidth, low latency, high reliability, and a massive number of connections, make it a great match for smart grid services. As a result, there is considerable interest in exploring how 5G can be applied in the context of smart grid technology [6].

In recent years, there has been an enormous growth in micro-electromechanical systems (MEMS) technology, leading to an increase in the use of low-cost sensors with advanced communication and data processing capabilities in the traditional Internet of Things (IoT). These wireless sensors require less power and can perform multiple functions, making them ideal for various applications such as health monitoring, security, environmental monitoring, industrial sensing, disaster prediction and diagnostics, and context-aware applications such as smart homes [6].

In this regard, the integration of 5G-enabled IoT in edge computing smart grids and the Metaverse can indeed enhance the efficacy of energy management. Smart grids can use sensors and meters to collect real-time data on energy consumption and generation by different nodes, which can then be analyzed and used to optimize the distribution and utilization of energy [3,30]. The use of IoT infrastructure can also enable the remote control of energy consumption and generation devices, allowing for more efficient energy usage. The Metaverse, which is an immersive virtual world, can also play a role in energy management by providing users with an interactive platform to monitor and manage their energy consumption. Through the use of smart devices and IoT infrastructure, users can control their energy usage from within the Metaverse, creating an immersive experience while also reducing their energy costs. The integration of IoT in smart grids and the Metaverse can also benefit energy providers, who can use the real-time data collected by IoT sensors to optimize their energy production and distribution processes. This can lead to the more efficient use of resources, reduced energy waste, and lower costs for both providers and end-users.

These advancements have led to the emergence of 5G-enabled IoT, which connects a wide range of objects to the internet including smartphones, PCs, smartwatches, and cars. With the use of 5G technology, these objects can transfer and process large amounts of data quickly and efficiently, making them more useful and practical for a variety of tasks. Additionally, the use of 5G-enabled IoT has given rise to new applications and services, such as autonomous vehicles and remote healthcare, that were previously not possible [6]. The birth of the second industrial revolution was marked by the rapid advancement of electrical engineering in the early 19th century, which replaced steam power with electrical energy and significantly impacted human development. Electricity is incredibly versatile and can be applied to various fields, including transportation, lighting, heat, telecommunications, and computers. Today, there are numerous technologies, such as hydroelectricity, solar power, wind power, and coal power, that contribute to the production of electric energy [31]. These energy sources are connected to create grid systems that deliver electricity to cities, businesses, and homes for daily life and work. Electrical energy has now become the foundation of all modern technologies. To ensure the safe, reliable, and energy-efficient operation of the grid, a range of grid management applications have been suggested. However, one of the significant challenges in monitoring and controlling grids is service response time. Recently, smart grid management applications based on the IoT and edge computing have been proposed to address this issue [31].

**Figure 1:**
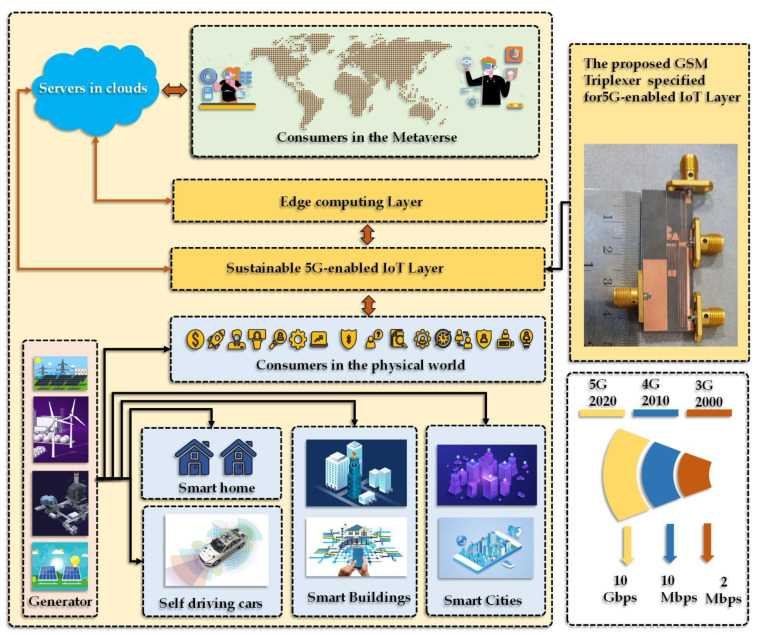
Proposed framework for enabling immersive interactions among diverse consumers. The framework incorporates cloud servers, edge computing layers, 5G-enabled IoT technology, and a triplexer to facilitate seamless communication between various types of consumers. The integration of the metaverse and the smart grid network enables the efficient management of complex systems.

Figure 1 depicts a proposed framework for enabling immersive interactions among various types of consumers, including self-driving cars, homes, buildings, and smart cities. The upper part of the figure represents how these consumers can interact with each other through the use of cloud servers. The cloud servers act as a central hub for storing, computing, and transferring information between consumers.

To increase the processing speed and reliability of the system, the proposed framework also incorporates edge computing layers. These edge computing layers are embedded in the system and provide additional computational resources closer to the consumers, reducing latency and increasing the overall system efficiency. The framework also includes a triplexer, which enables seamless communication between the different types of consumers. The triplexer is a component that allows the system to support multiple modes of communication simultaneously, such as voice, data, and video. This allows the various types of consumers to communicate with each other seamlessly, without the need for additional infrastructure or communication protocols. The use of 5G-enabled IoT technology ensures that communication between physical nodes and edge layers is secure and useful. Moreover, 5G technology offers high-speed data transmission, low latency, and reliable connectivity, making it an ideal choice for supporting the proposed framework.

Additionally, the figure illustrates how generators in the smart grid network are controlled by instructions produced in the metaverse. The metaverse is a virtual space where users can interact with each other and digital objects in a simulated environment. By integrating the metaverse with the smart grid network, the proposed framework can manage complex systems in a holistic manner, allowing for more efficient and effective control of the overall system. In summary, Figure 1 provides a comprehensive overview of the proposed framework’s components and their interactions. The use of cloud servers, edge computing layers, 5G-enabled IoT technology, and a triplexer allows for seamless communication between various types of consumers, while the integration of the metaverse and the smart grid network allows for efficient management of complex systems. Overall, this framework has the potential to enable immersive interactions and improve system efficiency in a variety of consumer applications.

## 3. Structure Analysis of the Proposed GSM Triplexer for 5G-Enabled IoT

**Figure 2:**
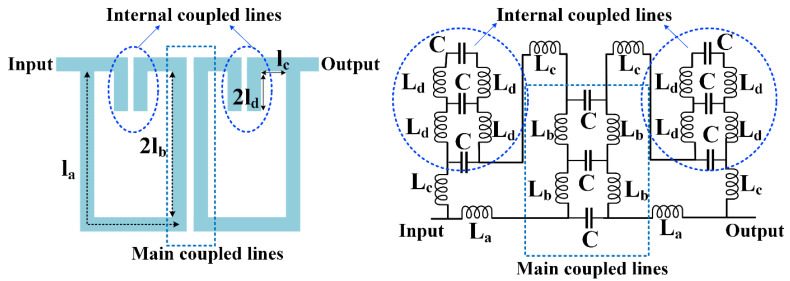
The proposed resonator and its approximated LC circuit.

The proposed resonator and its approximated LC circuit (a circuit which includes inductors and capacitors) are presented in Figure 2. Our resonator includes three pairs of coupled lines. The capacitors created by the coupling structure (*C*) effectively achieve the bandpass frequency response. First, we needed to generate a passband by introducing a novel resonator. To create a passband, we needed an equivalent structure with some inductors and capacitors. Using the coupled lines was a good choice because the equivalent LC of them can create a passband. To save the size, they can be embedded in two open loops. Finally, we reached the basic structure of the proposed resonator in Figure 2. By developing this structure using the proposed mathematical analysis, the desired triplexer with a novel structure could be achieved. In the LC model, we ignored the effects of bents, since they are effective at frequencies above 10 GHz, whereas our goal was to improve frequencies below 10 GHz [32]. The transmission lines with physical lengths l_a_, l_b_, l_c,_ and l_d_ were replaced by the inductors L_a_, L_b_, L_c,_ and L_d_, respectively. The equivalent of coupled lines was approximated, while in the more accurate model we have to increase the number of inductors and capacitors.

It is possible to simplify the LC circuit by replacing the equivalent impedance of internal coupled lines *Z*_1_, as follows:

(1) $$\begin{array}{l} Z_{1}=\frac{([\frac{(\frac{1}{j\omega C}+2j\omega L_{d})\times \frac{1}{j\omega C}}{\frac{1}{j\omega C}+\frac{1}{j\omega C}+2j\omega L_{d}}]+2j\omega L_{d})\times \frac{1}{j\omega C}}{[\frac{(\frac{1}{j\omega C}+2j\omega L_{d})\times \frac{1}{j\omega C}}{\frac{1}{j\omega C}+\frac{1}{j\omega C}+2j\omega L_{d}}]+2j\omega L_{d}+\frac{1}{j\omega C}}\Rightarrow Z_{1}=\frac{1-6\omega^{2}L_{d}C+4\omega^{4}C^{2}L_{d}^{2}}{8\omega^{3}L_{d}C^{2}-4\omega^{5}C^{3}L_{d}^{2}-3\omega C}j \end{array}$$

**Figure 3:**
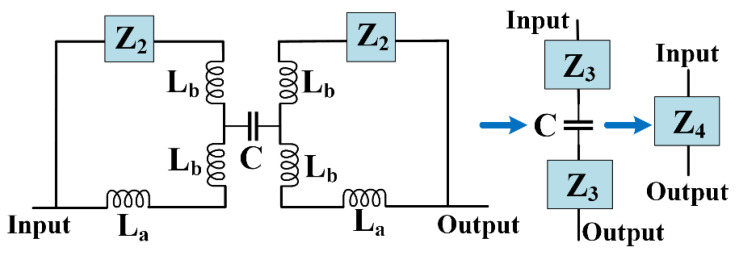
Simplified LC circuit.

In Equation (1), *ω* is an angular frequency. For an easy calculation, we have to use some approximations. We can set only the middle capacitor in the equivalent model of main coupled lines. Although the accuracy of this model was a little lower, we inevitably used this approximation to simplify very complex mathematical calculations. The simplified LC circuit is presented in Figure 3, where the impedance *Z*_2_ can be calculated as follows:

(2) $$Z_{2}=Z_{1}+2j\omega L_{c}\;\Rightarrow Z_{2}=\frac{1-6\omega^{2}L_{d}C+4\omega^{4}C^{2}L_{d}^{2}}{8\omega^{3}L_{d}C^{2}-4\omega^{5}C^{3}L_{d}^{2}-3\omega C}j+2j\omega L_{c}$$

Additionally, the impedance *Z*_3_ can be obtained easily using the following equation:

(3) $$Z_{3}=\frac{\left(Z_{2}+j\omega L_{b})\times (j\omega L_{b}+j\omega L_{a}\right)}{Z_{2}+j\omega L_{b}+j\omega L_{b}+j\omega L_{a}}=j\omega \frac{\left(Z_{2}+j\omega L_{b})\times (L_{b}+L_{a}\right)}{Z_{2}+2j\omega L_{b}+j\omega L_{a}}$$

By substituting *Z_2_* in Equation (3), *Z*_3_ is changed as follows:

(4) $$\begin{array}{l} Z_{3}=j\omega \frac{\left(\frac{1-6\omega^{2}L_{d}C+4\omega^{4}C^{2}L_{d}^{2}}{8\omega^{3}L_{d}C^{2}-4\omega^{5}C^{3}L_{d}^{2}-3\omega C}j+2j\omega L_{c}+j\omega L_{b})\times (L_{b}+L_{a}\right)}{\frac{1-6\omega^{2}L_{d}C+4\omega^{4}C^{2}L_{d}^{2}}{8\omega^{3}L_{d}C^{2}-4\omega^{5}C^{3}L_{d}^{2}-3\omega C}j+2j\omega L_{c}+2j\omega L_{b}+j\omega L_{a}}\Rightarrow \\ Z_{3}=\frac{j\omega (L_{b}+L_{a})[1-3\omega^{2}C(2L_{d}+2L_{c}+L_{b})+4\omega^{4}C^{2}L_{d}(L_{d}+4L_{c}+2L_{b})-4\omega^{6}C^{3}L_{d}^{2}(2L_{c}+L_{b})]}{1-3C\omega^{2}(2L_{d}+2L_{c}+2L_{b}+L_{a})+4\omega^{4}C^{2}L_{d}(L_{d}+4L_{c}+4L_{b}+2L_{a})-4\omega^{6}C^{3}L_{d}^{2}(2L_{c}+2L_{b}+L_{a})} \end{array}$$

Finally, the equivalent impedance between input and output ports (*Z*_4_) is:

(5) $$\begin{array}{l} Z_{4}=2Z_{3}+\frac{1}{j\omega C}\Rightarrow \\ Z_{4}=\frac{2j\omega (L_{b}+L_{a})[1-3\omega^{2}C(2L_{d}+2L_{c}+L_{b})+4\omega^{4}C^{2}L_{d}(L_{d}+4L_{c}+2L_{b})-4\omega^{6}C^{3}L_{d}^{2}(2L_{c}+L_{b})]}{1-3C\omega^{2}(2L_{d}+2L_{c}+2L_{b}+L_{a})+4\omega^{4}C^{2}L_{d}(L_{d}+4L_{c}+4L_{b}+2L_{a})-4\omega^{6}C^{3}L_{d}^{2}(2L_{c}+2L_{b}+L_{a})}+\frac{1}{j\omega C} \end{array}$$

The ABCD parameters of the proposed resonator and S-parameters are obtained using the following equation [21]:

(6) $$\begin{array}{l} A=D=1\;,\;B=Z_{4\;}\;\&\;\;C=0\\ S_{11}=\frac{A+B/Z_{0}-CZ_{0}-D}{A+B/Z_{0}+CZ_{0}+D}\;\;\;\;,\;\;\;\;S_{12}=\frac{2(AD-BC)}{A+B/Z_{0}+CZ_{0}+D}\\ S_{21}=\frac{2}{A+B/Z_{0}+CZ_{0}+D}\;\;\;\;,\;\;\;\;\;S_{22}=\frac{-A+B/Z_{0}-CZ_{0}+D}{A+B/Z_{0}+CZ_{0}+D} \end{array}$$

where *Z*_0_ is the impedance of terminals. According to the above equation, the scattering matrix (*S*) is defined by:

(7) $$S=\left[\begin{array}{l} S_{11\;}\;\;\;\;S_{12}\\ S_{21}\;\;\;\;\;S_{22} \end{array}\right]=\left[\begin{array}{l} \frac{Z_{4}}{2Z_{0}+Z_{4}}\;\;\;\;\;\;\;\;\;\;\;\;\frac{2Z_{0}}{2Z_{0}+Z_{4}}\\ \frac{2Z_{0}}{2Z_{0}+Z_{4}}\;\;\;\;\;\;\;\;\;\;\;\;\frac{Z_{4}}{2Z_{0}+Z_{4}} \end{array}\right]$$

For a lossless, passive two-port network |*S*_11_|^2^ + |*S*_21_|^2^ = 1, the condition for decreasing the loss can be found as follows:

(8) $${\left|\frac{Z_{4}}{2Z_{0}+Z_{4}}\right|}^{2}+{\left|\frac{2Z_{0}}{2Z_{0}+Z_{4}}\right|}^{2}=1\Rightarrow {\left|2Z_{0}+Z_{4}\right|}^{2}-{\left|Z_{4}\right|}^{2}=4Z_{0}^{2}\Rightarrow Z_{4}=0$$

The simulation results show that the coupling capacitors are usually small values in fF (or pF). Our target angular frequency (*ω*) is in GHz, and the inductors are in nH. Accordingly, we can use the following approximations to simplify Equation (5):

(9) $$\begin{array}{l} 1\rangle \rangle 3\omega^{2}C(2L_{d}+2L_{c}+L_{b})\;\;\;,\;\;\;\;1\rangle \rangle 3C\omega^{2}(2L_{d}+2L_{c}+2L_{b}+L_{a})\\ 1\rangle \rangle 4\omega^{4}C^{2}L_{d}(L_{d}+4L_{c}+2L_{b})\;\;\;,\;\;\;\;1\rangle \rangle 4\omega^{4}C^{2}L_{d}(L_{d}+4L_{c}+4L_{b}+2L_{a})\;\;\\ 1\rangle \rangle 4\omega^{6}C^{3}L_{d}^{2}(2L_{c}+L_{b})\;\;\;,\;\;\;\;1\rangle \rangle 4\omega^{6}C^{3}L_{d}^{2}(2L_{c}+2L_{b}+L_{a}) \end{array}$$

The following result will be obtained by combining Equations (8) and (9):

(10) $$Z_{4}\approx 2j\omega (L_{b}+L_{a})+\frac{1}{j\omega C}$$

Based on Equations (8) and (10), it can be written as:

(11) $$2j\omega (L_{b}+L_{a})+\frac{1}{j\omega C}=0\Rightarrow \omega =\sqrt{\frac{1}{2C(L_{b}+L_{a})}}$$

**Figure 4:**
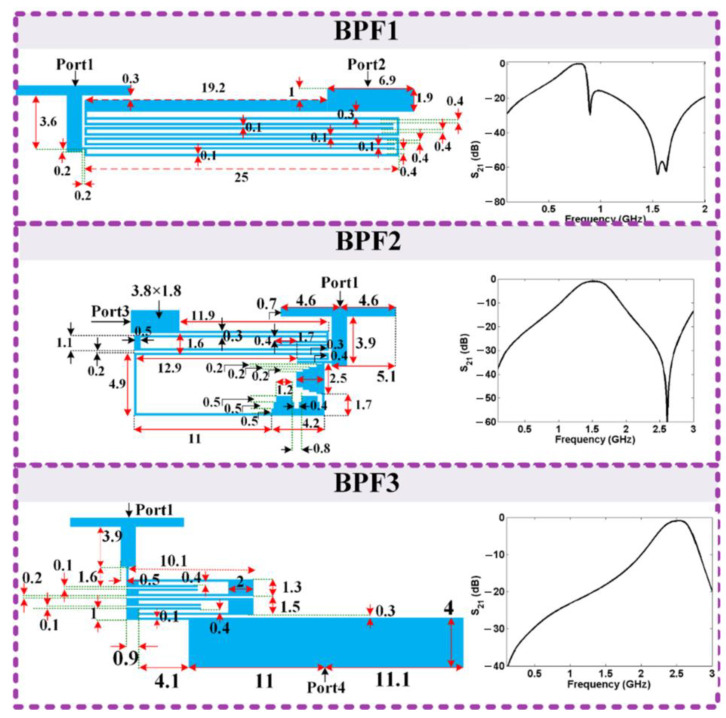
Layout and *S*_21_ of BPFs (unit: mm).

According to Equation (11), the values of inductors and coupling capacitors can be obtained for a predetermined angular frequency. Having the values of the inductors makes it possible to find the dimensions of the transmission lines using the Richards transformation [33]. Another important point is that the L_c_ inductor is less important in setting the resonant frequency and achieving low losses. However, without approximation the value of this inductor would appear in Equation (11). In total, by using the last equation it is possible to simultaneously adjust the resonance frequency and apply the necessary conditions to reduce losses. Using the analyzed resonator, three BPFs were designed. Figure 4 illustrates the BPFs with their frequency responses, where all dimensions are in mm. The widths of all thin lines were 0.1 mm. The frequency responses were obtained by Advanced Design Systems using an EM simulator with 0.005 GHz linear steps. A Rogers_RT_Duroid5880 substrate with a dielectric constant of 2.22, h = 0.7874 mm and tan(δ) = 0.0009 was used to design our filters and triplexer.

**Figure 5:**
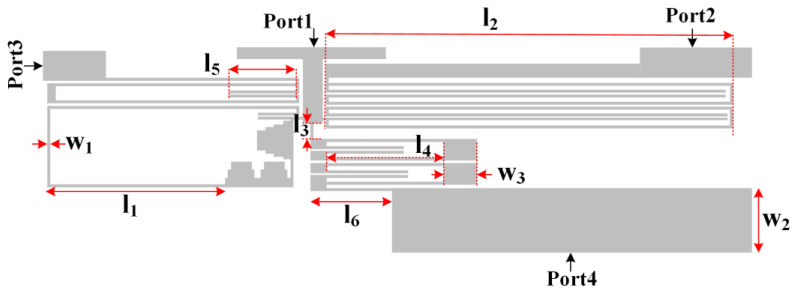
The layout of the proposed triplexer.

By integrating the BPFs, our triplexer was obtained as presented in Figure 5, where all dimensions were exactly equal to the corresponding filters. The overall size of our triplexer was equal to 43.7 mm × 12.7 mm = 0.157 λ_g_ × 0.045 λ_g_ = 0.007 λ_g_^2^, where λ_g_ is the guided wavelength calculated at 0.815 GHz.

**Figure 6:**
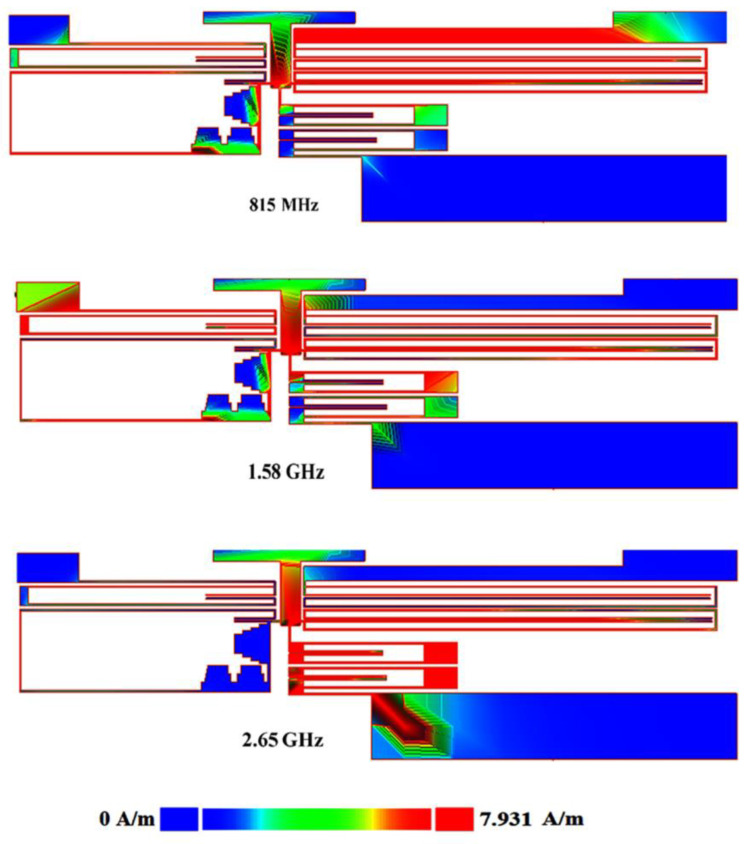
Current density distribution of our triplexer.

Figure 6 shows the current density distribution of the proposed triplexer for simulating ports 2, 3, and 4 at 0.815 GHz, 1.58 GHz, and 2.65 GHz, respectively. As shown in Figure 5, the wide sections (low-impedance sections) have lower current density [34]. According to the information obtained from the current density distribution, we can find the more effective lengths and widths for optimizing.

**Figure 7:**
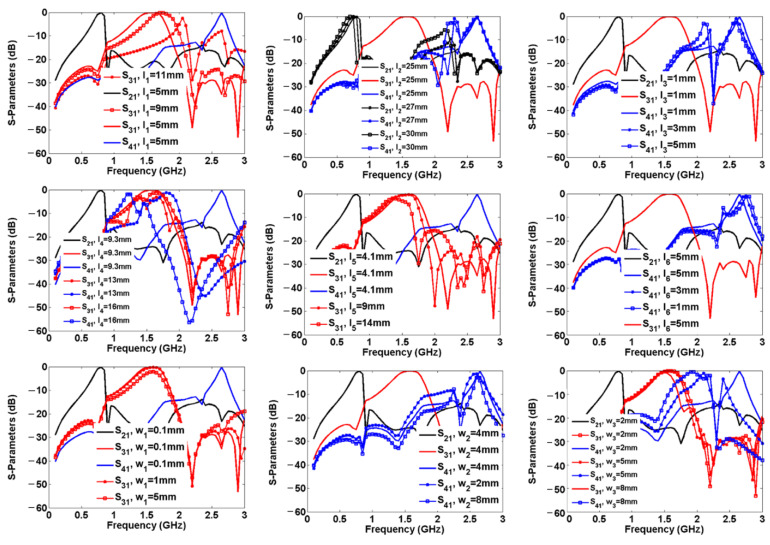
Frequency response as 9 functions of lengths and widths.

Hence, the frequency response as several functions of significant lengths and widths is depicted in Figure 7. As shown, the middle channel is impacted by changing the physical lengths l_1_, l_4_, l_5_, w_1,_ and w_3_, while the lower channel is only impacted by changing l_2_. By increasing the physical length l_2_ and width w_3_, some harmonics will appear. Increasing the length l_3_ leads to the appearance of harmonics created by BPF3. Also, decreasing l_6_ results in the appearance of the harmonics. However, the width of w_2_ should be neither large nor small, to suppress the harmonics.

**Figure 8:**
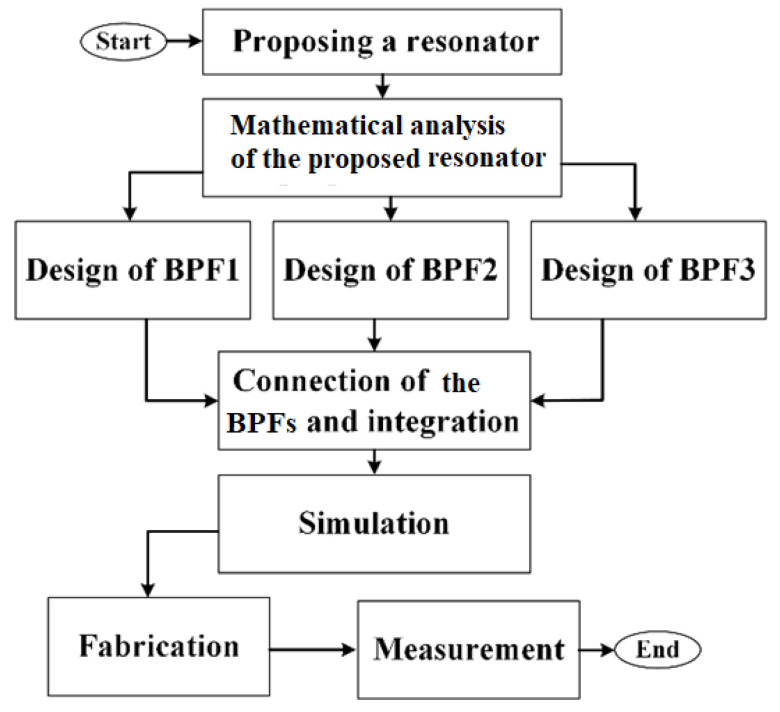
A flowchart of the triplexer design.

A flowchart for summarizing the triplexer design steps is shown in Figure 8. As shown in this figure, first, a suitable resonator was proposed. Then, it was analyzed mathematically. Using the analyzed resonator, three BPFs were designed and integrated to obtain a high-performance triplexer. After that, it was simulated. Finally, to verify the simulation results, it was fabricated and measured.

## 4. Results and Comparison

The simulation results were extracted from Advanced Design Systems (EM simulator). The measurement and simulation results were in good agreement where the measured results were obtained by an HP8757A network analyzer. HP8757A is a 20 GHz Network Analyzer from Agilent Company. It is a microprocessor-based receiver capable of making scaler (magnitude only) reflection and transmission measurements over a frequency range determined by the external detectors used. It has the following features:

- 10 MHz to 20 GHz measurement range;
- 76 dB dynamic range;
- Accurate swept power measurements;
- 40 dB directivity bridges;
- Four independent display channels;
- Limit testing built in;
- Save/recall setup and data;
- Direct plotter or printer output.

**Figure 9:**
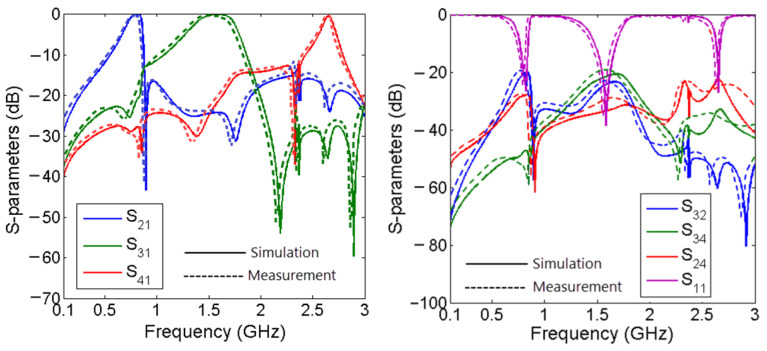
Simulated and measured frequency responses.

Figure 9 shows the simulated and measured frequency responses. The operating frequencies were F_O1_ = 0.815 GHz, F_O2_ = 1.58 GHz, and F_O3_ = 2.65 GHz, with three low insertion losses of 0.12 dB, 0.09 dB, and 0.42 dB, respectively. The first and second channels were wide, with 20% and 27.2% fractional bandwidths (FBW_1_ and FBW_2_), respectively. The common port return losses in the first, second, and third channels was better than 26.6 dB, 38.4 dB, and 27.1 dB, respectively. The measured losses were a little more than simulations due to copper and SMA losses. As shown in Figure 9, the isolations between channels (*S*_23_, *S*_24_, and *S*_34_) were better than −20 dB. The RLs from ports 2, 3, and 4 (*S*_22_, *S*_33,_ and *S*_44_) were better than 27.7 dB, 45.1 dB, and 21.7 dB, respectively.

**Figure 10:**
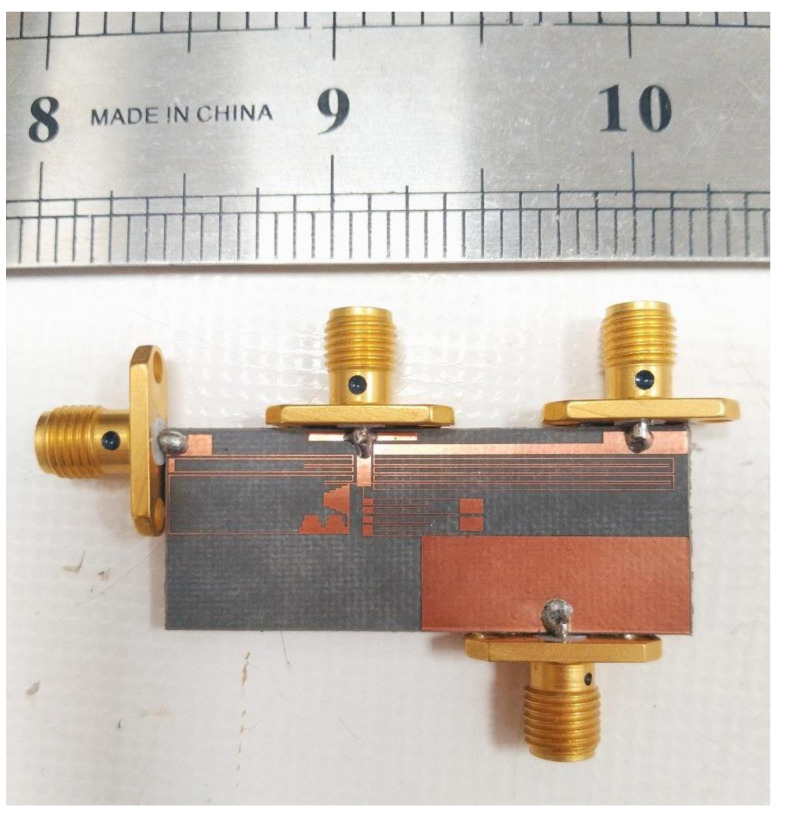
A photograph of the fabricated triplexer.

The measured insertion losses were 0.39 dB, 0.21 dB, and 0.66 dB at the first, second and third passbands, respectively. The measured isolation between the output ports was better than −19.5 dB. The measured common port return losses at the lower, middle and upper channels were better than 24 dB, 35.1 dB, and 24.1 dB, respectively. From these results, it is clear that the proposed triplexer has high performance compared to the previous designs. A photograph of the fabricated triplexer is presented in Figure 10.

To show the features of this triplexer, we compared it with previously reported microstrip triplexers in terms of size and performance. The comparison results are presented in Table 2, where IL_1_, IL_2,_ and IL_3_ were the insertion losses at the first, second, and third channels, respectively. The RLs at the first, second, and third channels were presented by RL_1_, RL_2,_ and RL_3_, respectively. Since the design of a microstrip triplexer is more complicated and difficult than microstrip filters and diplexers, there are not many recent triplexers to compare with our design. The actual performance gain in the triplexer design depends on various factors including novel structure, designing process, compact size, low losses, high isolation, wide FBW, and agreement between the mathematical analysis, simulation, and experimental results. Therefore, it is fair to compare the performance of triplexers with the parameters presented in our comparison table. These are the same parameters that the previous works mentioned to compare and show their superiority.

The comparison results show that our triplexer had the most compact size, with the lowest insertion losses at all channels. Moreover, the widest FBWs and the best common port return losses at the first and second channels were obtained in this work. According to the comparison Table, the size of our proposed triplexer was only 0.007 λ_g_^2^, while no triplexers with a size smaller than 0.01 λ_g_^2^ have been reported yet. We obtained this achievement without any negative effects on the frequency responses. As shown in Table 2, we could minimize the losses while keeping our triplexer bandwidth reasonable. The proposed structure is novel and it is presented for the first time in this work. Its physical structure is not similar to the other previous structures. In addition, the obtained return losses are good. As can be seen, the measurement (experimental) results confirm the mathematical analysis and simulation results. Therefore, our triplexer is a novel design and has high performance compared to the previous works. Table 3 presents the parameters and components used to design the proposed triplexer.

## 5. Discussion

Currently, the Metaverse is considered a trending topic in numerous research areas and is currently the fastest-growing sector in high-tech [36,37]. The significance of data processing and interpretation is increasing, including for engineers, as a result of energy digitalization. Smart grids refer to several new data-driven services in usage, storage, marketing, and renewable energy supply [30]. This paper presents a highly efficient GSM triplexer for 5G-enabled IoT in smart grid edge computing and the Metaverse based on recent developments and fundamental ML methods in energy distribution. The 5G-enabled IoT helps in the quick and efficient handling of data in energy distribution, including big data analytics, energy devices, and materials, transmission and distribution infrastructure, individual consumers, conventional grid or smart grid infrastructure, and transmitting energy from transmission systems. Firstly, we recognized the multitude of demands that smart grids and the Metaverse aim to overcome, scrutinize recent developments in the field, and assessed the impact of 5G-enabled IoT on the energy industry. We provided a conceptual framework that can be used to tackle intricate problems. Furthermore, we delved into different facets of IoT applications in essential energy technologies and briefly discussed five instances where IoT is utilized in energy distribution. Additionally, we highlighted the infrastructural requirements for 5G-enabled IoT in energy distribution, the challenges involved, opportunities for the Metaverse towards a smart and sustainable future, and the recent advancements in discovering and understanding models, all of which reinforce the main focus of this research.

In this respect, we designed and evaluated a triplexer component that is super-efficient and intended for use in 5G-enabled IoT in sustainable smart grid edge computing and the Metaverse. The proposed triplexer is designed and fabricated to operate at the specific frequencies of 0.81 GHz for the low-band 5G applications and 1.58 GHz and 2.65 for the mid-band 5G applications. One of the key features of our proposed triplexer is its small size, which is only 0.007 λ_g_^2^, making it significantly smaller than reported microstrip triplexers, which typically could not achieve a size less than 0.01 λ_g_^2^. Despite its compact size, our triplexer has good performance, with very low insertion losses at the first, second, and third channels, which are 0.12/0.09/0.42 dB, respectively. The common port return loss (RL) at the first, second, and third channels is good, and better than 26/38/27 dB, respectively. To achieve this good performance, we designed a resonator consisting of three pairs of coupled lines that were mathematically analyzed to tune the resonance frequency and reduce losses. We also optimized the physical dimensions of the triplexer to further improve its performance.

Overall, the proposed triplexer is super-efficient and small in size, making it an attractive option for use in 5G-enabled IoT in sustainable smart grid edge computing and the Metaverse. The good performance of our triplexer makes it suitable for use in a variety of applications where efficient signal transmission and low losses are critical.

This 5G infrastructure, particularly 5G-enabled IoT, has great potential to enhance the profitability and reliability of investors and power companies, contributing to greater sustainability and facilitating the transition to cleaner energy sources. Although there are still various ways in which IoT devices can be deployed, they can expedite the energy industry’s positive transition and contribute to reducing carbon emissions.

Ensuring an uninterrupted energy supply to consumers is a significant challenge for the power grid, one which is influenced by various factors such as interferences, internal faults, unpredictable incidents, weather patterns, and energy consumption [2]. To overcome these challenges, power grids increasingly adopt sustainable networks equipped with advanced technologies and IoT components to optimize their performance. The IoT is being implemented in various energy distribution applications, and power utilities and grid operators use it to connect grid failure conditions at the distribution, transmission, and generation levels. Sensors and smart meters such as phase measurement units provide real-time energy consumption data. By integrating smart grid infrastructures and the Metaverse with optimized IoT components, we can enhance the immersive interactive experience and achieve better performance. Furthermore, recent advancements in industrial informatics suggest that 5G-enabled IoT can facilitate and improve the deployment and advancement of advanced energy materials and electric infrastructure at the energy distribution level.

The integration of 5G-enabled IoT in edge computing smart grids and the Metaverse can improve energy management. Smart grids can gather real-time data on energy consumption and generation from different nodes through sensors and meters. These data can be analyzed to enhance energy distribution and utilization [6]. The use of IoT infrastructure can also allow for the remote control of energy devices, which can lead to more efficient energy usage. The Metaverse, a virtual world, can also contribute to energy management by providing an interactive platform for users to monitor and manage their energy consumption using smart devices and IoT infrastructure. This integration can also benefit energy providers, who can optimize their energy production and distribution processes with real-time data from IoT sensors, resulting in more efficient resource utilization, reduced energy waste, and lower costs for both end-users and providers [3].

The integration of IoT technology in smart grids offers boundless opportunities. This allows utility providers to connect with consumers seamlessly through precise communication, leading to increased productivity in a simplified manner. The IoT facilitates social collaboration, resulting in swift decision-making and a reduced total cost of ownership of applications. This interconnectivity is enabled through mobile phone communication. Cloud technology provides significant financial benefits, especially when considering the total cost of ownership of a particular solution, including its impact on services, operating expenses, and purchase costs. By investing in improving their product services, companies can secure stable pricing for the duration of a contract period without having to worry about hidden costs [38].

In summary, microstrip components such as the suggested Global System for Mobile Communications (GSM) triplexer provide a range of advantages for sustainable IoT in smart grids and the metaverse. The most significant advantage of microstrip technology is that it only requires a two-layer board, with all components mounted on a single side, making it the most cost-effective RF circuit board solution [39,40]. As all components are located on the same surface, the use of vias when making connections is unnecessary, avoiding the introduction of unwanted capacitance or inductance. Microstrip traces allow for better control over the characteristic impedance of the trace, making it easier to maintain the intended impedance. Due to the wider width of microstrip traces, the etching tolerances in fabrication are absolute values, minimizing the impact of over-etching on the trace width and the resulting characteristic impedance. This is in contrast to stripline traces, which are more sensitive to changes in width due to over-etching. The ability to control characteristic impedance is crucial in RF circuit board design as it ensures the circuit operates within the intended frequency range. Additionally, some designs specify the finished trace width rather than the characteristic impedance of the trace, and microstrip traces offer greater tolerance for changes in width due to over-etching than stripline traces. Microstrip components provide cost-effective, efficient, and precise solutions for sustainable IoT in smart grids and the metaverse. Their two-layer board design, control over characteristic impedance, and simplified fabrication and assembly processes make them an excellent choice for RF circuit board design.

Microstrip components are an ideal option for building a sustainable 5G network and can significantly improve the resilience of the network in case of failures. Their cost-effectiveness, efficient design, and precise control over characteristic impedance make them a reliable solution for RF circuit board design, especially in high-frequency applications such as 5G networks.

Microstrip components have become a popular choice for sustainable smart grid edge computing due to several benefits. These components are energy-efficient, consuming less power than traditional components, which is important in edge computing, where power consumption is a significant concern. In addition, microstrip components have a small form factor, making them suitable for use in edge computing devices that have limited physical space. Furthermore, microstrip components are highly customizable and can be designed to meet specific requirements, which is important for smart grid edge computing systems that need to meet specific performance criteria. Lastly, microstrip components can be produced using low-cost manufacturing techniques, making them more affordable than traditional components, which can help to reduce the overall cost of smart grid edge computing systems and promote sustainability. Overall, microstrip components offer several advantages that make them well-suited for sustainable smart grid edge computing, promoting energy efficiency and reducing environmental impact.

## 6. Conclusions

The increasing need for energy and the effects of climate change have caused concerns about energy sustainability and environmental conservation worldwide. The development of smart grid technology has shown that traditional private power networks are inadequate for meeting the demands of industrial applications. The growth of the 5G network industry will heavily rely on the Internet of Things (IoT) for further progress, which will drive the expansion of future smart grids. This advanced platform will not only provide the communication infrastructure for smart grid edge computing, but will also include Metaverse platforms. Therefore, optimizing the IoT is essential for creating a sustainable edge-computing network. This article introduces the new design, manufacturing, and evaluation of an ultra-efficient GSM triplexer specifically created for 5G-enabled IoT in sustainable smart grid edge computing and the Metaverse. The proposed resonator is examined to acquire a scattering matrix and identify the condition for minimizing loss. The triplexer presented in the paper operates at 0.81 GHz (for low-band 5G and GSM), 1.58 GHz (for mid-band 5G), and 2.65 GHz (for mid-band 5G). The advantages of this triplexer are low insertion and return losses at all channels, wide fractional bandwidths at the first and second channels (20% and 27.2%), and a compact size of 0.007 λ_g_^2^ = 555 mm^2^.

## Figures and Tables

**Table 1:** The features of the previous triplexers (WLANs: Wireless Local Area Networks, WiMAX: Worldwide Interoperability for Microwave Access).

Refs.	Basic Resonator	Applications	Advantages	Disadvantages
[13]	Coupled meandrous lines	GSM, WLANs	Wide channels	Large size, high losses
[14]	Star-junction topology	WiMAX	Attenuated harmonics	Large size, high losses
[15]	Asymmetric split-ring	WiMAX	---	Large size, high insertion loss
[16]	Parallel coupled line	GSM	High isolation	Large size, high insertion loss, narrow channels
[17]	Common triple mode	GSM	---	Large size, high insertion loss, narrow channels
[18]	Multimode net type	Wireless	High selectivity	Large size, high losses
[19]	Coupled step impedance	4G, WiMAX	Low losses	Large size
[20]	Zigzag coupled lines	GSM, WLANs	---	Large size
[21]	Open loop	GSM, WiMAX	Good return loss	Large size, high insertion loss
[22]	Coupled lines	GSM, WiMAX	Good return loss	Large size, high insertion loss
[23]	Quarter-wave	GSM, WiMAX	Good return loss	Large size, high insertion loss

**Table 2:** Comparison between our proposed triplexer and the previous works (*: approximate value).

Refs.	F_O1_/F_O2_/F_O3_(GHz)	IL1/IL2/IL3(dB)	RL1/RL2/RL3(dB)	FBW1, FBW2, FBW3	Size (λ_g_^2^)
This Triplexer	0.81/1.58/2.65	0.12/0.09/0.42	26.6/38.4/27.1	20%, 27.2%, 5%	0.007
[3]	3.2/3.7/4.4	2.7, 2.5, 1.8	16/16/16	6.5%, 7%, 8%	0.048
[15]	1.2/1.8/2.4	1.3/1.3/1.2	11.6/14/10	14.4%, 14%, 13.6%	0.055
[16]	3.3/3.89/4.56	2.2/2.3/2.3	Better than 14	---	0.275
[17]	2.15/2.95/3.8	2.2/1.9/1.7	Better than 20	---	0.0164
[18]	1.5/1.7/1.9	4.9/5.8/5.95	---	3.3%, 2.9%, 3.6%	0.132
[19]	1.88/2.1/2.6	1.3/2.3/3.2	22/25/21	0.86%, 1.4%, 0.96%	0.1 *
[20]	1/1.25/1.5	2.7/1.8/3.2	Better than 16	9.5%, 4.2%, 4.5%	0.064
[21]	2.67/3.1/3.43	0.72/0.63/0.71	24.5/24/24.7	---	0.137
[22]	0.9/2.4/5.5	0.7/1.7/1.5	---	---	---
[23]	1.8/3.2/4.4	1.97/1.99/2.3	24/22/25	7.44%, 7.45%, 6.2%	0.177
[24]	1.4/1.8/3.2	0.1/2/1	25/20/20	5.2%, 2.8%, 9.4%	0.014
[25]	1.75/2.35/3.68	1.3/1.4/1.7	20/25/30	5.7%, 8.5%, 6.8%	0.027
[26]	1.45/2.15/2.75	3.6/4.3/4.8	15/20/15	6%, 6%, 4%	0.020
[27]	2.4/3.5/5.2	2.42/1.62/1.95	Better than 15	3%, 7%, 3%	0.164
[28]	2.3/3.2/3.6	0.78/1.1/0.62	19.8/10/28	5.2%, 5.5%, 1.6%	0.095
[29]	2.05/2.45/3.5	1.5/1.8/1.5	Better than 13	4.8%, 4%, 5.7%	0.346
[35]	2.1/2.5/3	1.4/1.8/1.6	---	---	0.052

**Table 3:** Parameters and components used to design the proposed triplexer.

Substrate	Rogers RT/Duroid 5880
ε_r_	2.22
h	0.7874 mm
tan(δ)	0.0009
The software used to obtain the simulation results	Advanced Design Systems (ADS)
The device used to measure the experimental results	HP8757A network analyzer
Dimensions	Exactly like the proposed BPFs

## Data Availability

Not applicable.
